# Dynamics and recognition of glycosphingolipids in membranes: insights from different scales

**DOI:** 10.1007/s12551-026-01430-1

**Published:** 2026-03-30

**Authors:** Olga N. Makshakova, Winfried Römer

**Affiliations:** 1 Faculty of Biology, Synthetic Biology of Signalling Processes Laboratory, University of Freiburg, 79104 Freiburg, Germany; 2 Signalling Research Centres BIOSS and CIBSS, University of Freiburg, 79104 Freiburg, Germany; 3 Kazan Institute of Biochemistry and Biophysics, FRC Kazan Scientific Center of RAS, 420111 Kazan, Russia

**Keywords:** Glycolipids, Lectins, Synthetic membrane systems, Advanced microscopy techniques, Molecular dynamics, Computer simulations

## Abstract

Glycosphingolipids are the most complex molecules among mammalian lipids. They consist of a ceramide backbone and a glycan head, so their description combines the challenges of lipids and carbohydrates. The carbohydrate moiety (with high conformational flexibility) serves as a receptor for carbohydrate-binding proteins, e.g., lectins, including molecules of pathogenic origin. Glycosphingolipids exhibit inhomogeneous lateral diffusion, which supports the formation of nanoscale functional complexes during signal transduction. These complexes enriched with glycosphingolipids can also contain GPI-anchored proteins, transmembrane proteins, and phospholipids from both membrane leaflets via acyl chain interdigitation, thereby transmitting the signal from outside the cell to the cell interior. The formation process of such molecular complexes extends over a wide spatial and temporal range and still remains largely unexplored due to the resolution limits of existing observation techniques. In this review, we bring together insights into the dynamics of glycosphingolipids in membranes and their interactions with carbohydrate-binding proteins from high/super-resolution fluorescence microscopy and molecular modelling—illustrated with examples such as the gangliosides GM1 and GM3, and the globoside globotriaosylceramide (also known as Gb3, CD77, and P^k^ antigen)—to exemplify how the gap in spatial and temporal resolution between experimental and computational approaches is steadily narrowing. The selected case studies present investigations using both cells and membrane model systems, such as giant unilamellar vesicles and supported lipid bilayers, together with *in silico* lipid bilayers with the aim of reducing the environmental complexity to determine the key molecular determinants involved in functional complex formation, membrane bending, endocytosis, and signalling.

## Introduction

Glycosphingolipids (GSLs) play diverse and essential roles in health and disease (Celi et al. 2022; Nicoli et al. 2021; Chiricozzi et al. 2020). As integral components of cell membranes, they are key determinants of cell identity orchestrating processes such as immune response, cell-cell communication, and neuronal function (Alves et al. 2022; Ekyalongo et al. 2015; Möckl 2020). The distribution of GSLs within membranes is heterogeneous, often contributing to transient dynamic micro- or nanoscale domains (Roy and Patra 2023; Hanafusa et al. 2020). They are mainly located in the outer (i.e., extracellular) leaflet of the plasma membrane and are often found together with glycosylphosphatidylinositol (GPI)-anchored proteins in sphingomyelin- and cholesterol-enriched domains commonly referred to as lipid rafts (Kinnun et al. 2023; Anselmo et al. 2025).

Due to the diverse glycosylation patterns of their headgroups, GSLs contribute to the organization of proteins, including growth factor receptors, within membrane domains (Wang and Yu 2021, 2013). By controlling the recruitment or exclusion of molecules, they modulate downstream signalling pathways, such as the activity of tyrosine kinase receptors (Russo et al. 2016; Brandel et al. 2021; Kovacs et al. 2022). As regards the lipid moiety of GSL, variations in the length and degree of saturation of the fatty acyl chain influence their lateral distribution within the membrane (Arumugam et al. 2021). These structural differences also affect interaction with lipids in the inner (i.e., cytosolic) leaflet of the plasma membrane and form the basis for transmembrane signal transduction through interleaflet coupling (Skotland and Sandvig 2019; Ewers et al. 2005).

### Presentation of glycosphingolipids on the membrane surface

The composition of the carbohydrate headgroup is crucial for the specific recognition of GSLs by carbohydrate-binding proteins, including lectins, from both hosts and pathogens (Mattox and Bailey-Kellogg 2021). Furthermore, the glycan moiety exhibits high conformational flexibility, allowing it to adapt to and bind to proteins with different arrangements of carbohydrate-binding sites. In this context, the recognition of the glycans can be imagined as the selection of a suitable “key” from a multitude of different conformations (referred to as a “bunch of keys”), in contrast to the traditional “lock-and-key” model (Siebert et al. 2003; Topin et al. 2016; Re et al. 2011). Within the local membrane environment, steric hindrance influences the distribution of glycan conformations by modulating their presentation and accessibility to carbohydrate-binding proteins. GSLs exhibit “crypticity,” a property that allows them to evade protein binding by dynamically modulating their topological presentation on the membrane surface (Chiricozzi et al. 2020; Lete et al. 2022; Greenshields et al. 2009). The molecular determinants that determine glycan presentation and accessibility to glycan-binding proteins include the length and structure of the fatty acyl chain (DeMarco 2012; Hamilton et al. 1994), interactions between the carbohydrate moieties of clustered GSL molecules, direct contacts with cholesterol and other components such as transmembrane or GPI-anchored proteins in the environment of plasma membrane (Lingwood et al. 2011; Novak et al. 2013; Watkins et al. 2014).

### Glycosphingolipids as targets for therapeutical agents

Dysregulation of GSLs due to altered abundance, truncation, or partial cleavage of the carbohydrate headgroup is the cause of important pathological features such as neuroinflammation and impaired signal transduction (Hülsmeier 2025). Consequently, it is a hallmark of diverse diseases, including lysosomal storage disorders such as Fabry and Gaucher disease (Zhou et al. 2022) as well as various types of cancer (Costa et al. 2020; García-Hevia et al. 2022; Haar Àvila et al. 2023). Lectins and antibodies that recognize specific carbohydrate patterns are valuable tools for mapping aberrant glycosylation on the cell surface (Islam et al. 2023; Soliman et al. 2017; Rodriguez et al. 2018). However, their effectiveness in detecting glycosphingolipids is limited by the accessibility of the carbohydrate headgroups (Novak et al. 2013; Meléndez et al. 2022; Volynsky et al. 2017). By using lectins that are specific for the host cell’s glycosphingolipid Gb3, it has been shown that lectin-based CAR-T cells and lectibodies recognize Gb3-positive cancer cell lines and induce their destruction (Meléndez et al. 2022; Rosato et al. 2022; Tomisch et al. 2023, 2025). However, the efficiency of the α-galactose-specific lectins used to recognize Gb3 varied considerably. Across different cancer cell lines, each lectin exhibited a marked preference for Gb3 species defined by their fatty acyl chain composition regardless of their relative abundance within the cells (Meléndez et al. 2022). In addition, three commercially available anti-Gb3 antibodies bound well, but to varying degrees, to the plasma membrane of Gb3-positive Ramos Burkitt lymphoma cells. In contrast, the same antibodies showed almost no binding to the cell surface of fibroblast cells from Fabry patients and H1299 lung epithelial cells, while the two Gb3-binding lectins, the B-subunit of Shiga toxin (StxB) from *Shigella dysenteriae* and LecA from *Pseudomonas aeruginosa* recognized these cells convincingly (Elçin-Guinot et al. 2025). In human tumor biopsies, the masking of tumor-associated GSLs (including Gb3) from immunodetection was attributed to interactions between their sugar moieties and cholesterol, as well as a potential tilt of the carbohydrate headgroup. In contrast, these GSLs became accessible for antibody recognition after cholesterol depletion (Novak et al. 2013). Another study reported that natural antibodies specific to the Gb3 trisaccharide (αGal1,4βGal1,4βGlc) did not bind to erythrocytes, regardless of the presence of cholesterol. However, the Gb3 trisaccharide became recognizable to the antibodies when a synthetic Gb3 analogue, a FSL (Function-Spacer-Lipid) construct, was integrated in the membrane, in which a chemical spacer of approximately 12 atoms was inserted between the Gb3 trisaccharide and the DOPE lipid moiety (Volynsky et al. 2017). Therefore, GSLs are promising but challenging targets for the disease-related detection. A deeper understanding of how these important structural and dynamical properties, such as the composition of fatty acyl chains and the flexibility of carbohydrate moieties, influence the distribution of glycosphingolipids in the plasma membrane and their accessibility to the binding partners will contribute to the development of new approaches for the selective mapping of target glycosphingolipids.

### Visualization of glycosphingolipids using imagine techniques and computer simulations

**Fig. 1:**
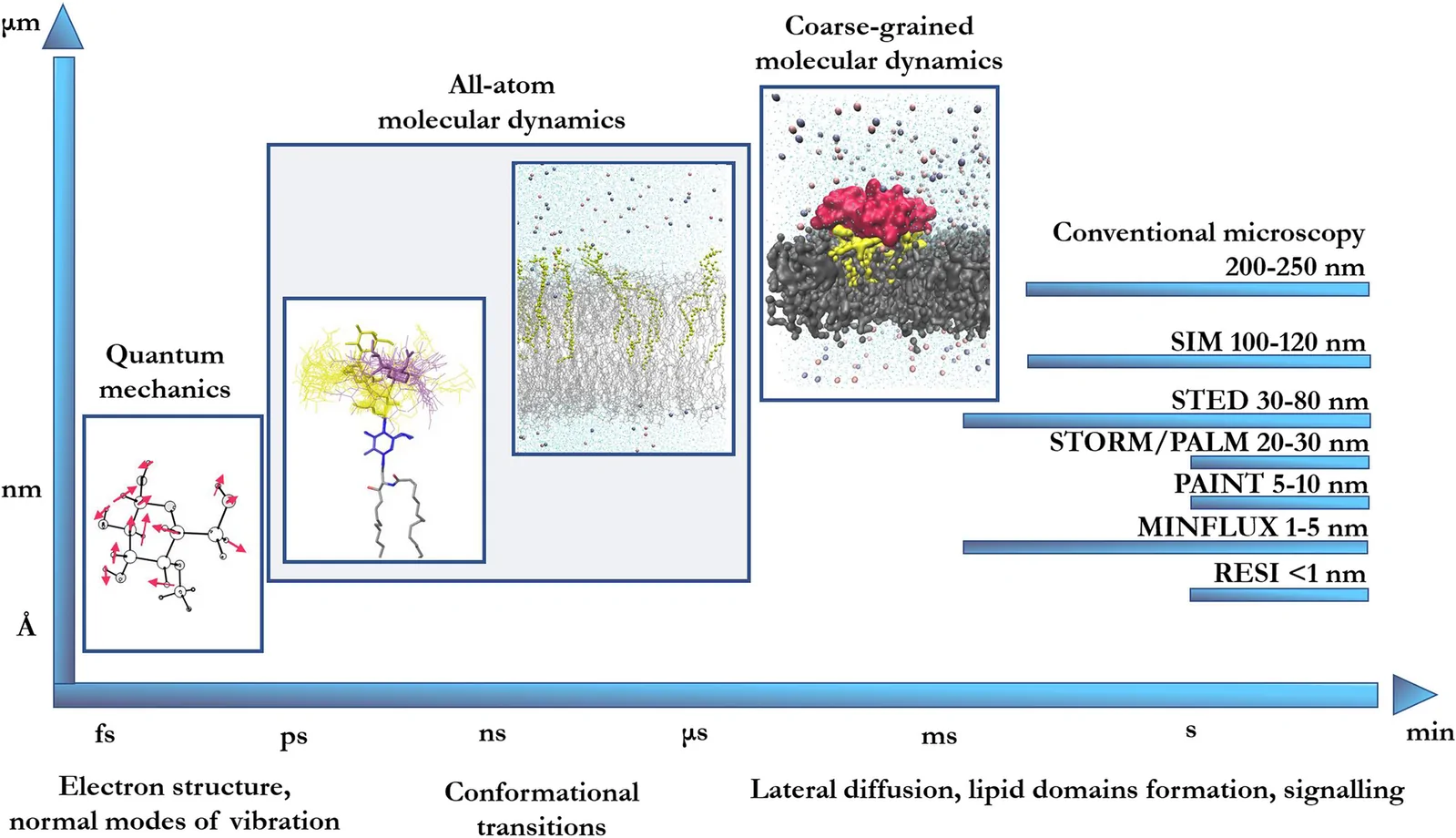
A schematic presentation of the spatial and temporal scales that can be accessed using microscopy techniques and computer modelling approaches

The processes described—lateral diffusion of glycosphingolipids, conformational dynamics, association and dissociation with proteins and transient membrane domain formation—span large spatial and temporal scales, making it difficult to study these aspects experimentally and theoretically in the biological context. Experimentally, the spatial resolution achieved by classical fluorescence microscopy techniques remains limited by hundreds of nanometers and does not allow to distinguish intermolecular interactions but rather evidences about a co-occurrence of the molecules in quite a large area with a temporal precision down to milliseconds. In contrast, *in silico* approaches provide atomistic details on intermolecular interactions and the dynamics of quickly relaxing motions, but the size of the membrane system is usually regulated by a compromise with the desired trajectory length (depending on the available computer resources) and is typically limited to microseconds. Modern methods of computer modelling (Perez and Makshakova 2022; Marrink et al. 2019) and super-resolution microscopy that overcome the diffraction limit are steadily closing this gap (Fig. 1) (Wu and Shroff 2022; Turkowyd et al. 2016). In this review, we provide an overview of fluorescent microscopy techniques and computer modelling approaches illustrated by several case studies on the dynamics of glycosphingolipids and complexes with proteins in the membrane context. The focus here is on two relatively well-studied GSLs: GM1, a ganglioside mainly abundant in neurons, and Gb3. The case studies encompass their interactions with raft-associated signalling proteins anchored into the plasma membrane and bacterial lectins. This review article does not claim to be comprehensive. The examples described are not sorted in the order of publication date, but rather according to their significance in highlighting the progress made to date and the limitations that still need to be overcome in visualizing GSL dynamics and deciphering the role of structural aspects of GSLs in their recognition by lectins and in intermolecular interactions with receptor proteins that lead to signal transduction.

## Fluorescence microscopy techniques for investigating the dynamics of glycosphingolipids in membranes

### Tracking of glycosphingolipids in membranes

Fluorescence microscopy is a robust tool for determining the cellular localization of a molecule carrying a fluorophore, and its proximity to other molecules based on the colocalization of fluorescent markers that emit at different wavelengths. Fluorescent synthetic glycolipids are particularly well suited for investigating the localization and dynamics of GSLs, as they not only enable the attachment of a fluorophore to the glycolipid, but also allow control of the backbone and the fatty acyl chain structure, the composition of the carbohydrate head, and, if necessary, the introduction of a linker between the carbohydrate headgroup and the lipid (Ryzhov et al. 2025), even if they no longer represent fully natural molecules. Synthetic glycolipids can be incorporated either exogenously into the cell plasma membrane or into minimalist artificial lipid bilayers such as giant unilamellar vesicles (GUVs) or supported lipid bilayers (SLBs) with controllable lipid composition (Madl et al. 2017). In addition to chemical synthesis, glycosphingolipids can also be partially built/modified enzymatically directly on the surface of membranes (Craven et al. 2018; Jabeguero et al. 2023). Among other things, this enables the incorporation of azido-sugars with subsequent labeling with imaging probes (Laughlin et al. 2006). In addition, fluorescently labeled lectins can be used to detect natural, i.e., non-fluorescent glycosphingolipids, even though some lectins can induce a local reorganization of molecules when used in living cells. For example, the B-subunits of cholera toxin (CTxB) and Shiga toxin (STxB) are highly specific probes for GM1 and Gb3, respectively.

### Spatial resolution of light microscopy

The resolution of optical microscopy is limited by the Abbe diffraction limit. The distance at which two individual dots can be distinguished is proportional to the wavelength of the light and inversely proportional to twice the numerical aperture of the objective lens. The resolution is far from sufficient to distinguish single molecules (~200 nm lateral resolution and ~ 800 nm axial resolution). Revolutionary breakthroughs in overcoming the diffraction limit in recent decades have led to the development of a number of super-resolution techniques, including structured illumination microscopy (SIM) (Guerra 1995), stimulated emission depletion (STED) microscopy (Hell and Wichmann 1994), and a family of single molecule localization microscopy (SMLM) techniques including photoactivated localization microscopy (PALM) and stochastic optical reconstruction microscopy (STORM) (Rust et al. 2006). SIM uses structured gratings that, through systematic shifting, produce a Moiré fringe pattern that can be reconstructed into images with higher resolution (~100 nm), thereby doubling the lateral resolution of a light microscope. STED uses a secondary donut-shaped beam to quench fluorescence at the periphery of the excitation area, thereby improving the resolution (~50 nm). SMLM is based on the random blinking of fluorophores, which allows a subset of fluorescent molecules to be localized in each illumination cycle. The position of the fluorophores can then be calculated, reaching resolutions of 20 nm. However, with improved spatial resolution, SMLM methods require more time to complete data acquisition (Fig. 1). For a more detailed discussion of super-resolution microscopy and its applications in the study of membrane nanodomains and lipid rafts, readers are referred to recent review articles (Anselmo et al. 2025; Zulueta Diaz and Arnspang 2024).

### Visualization of membrane dynamics induced by pathogenic Gb3-binding lectins

**Fig. 2:**
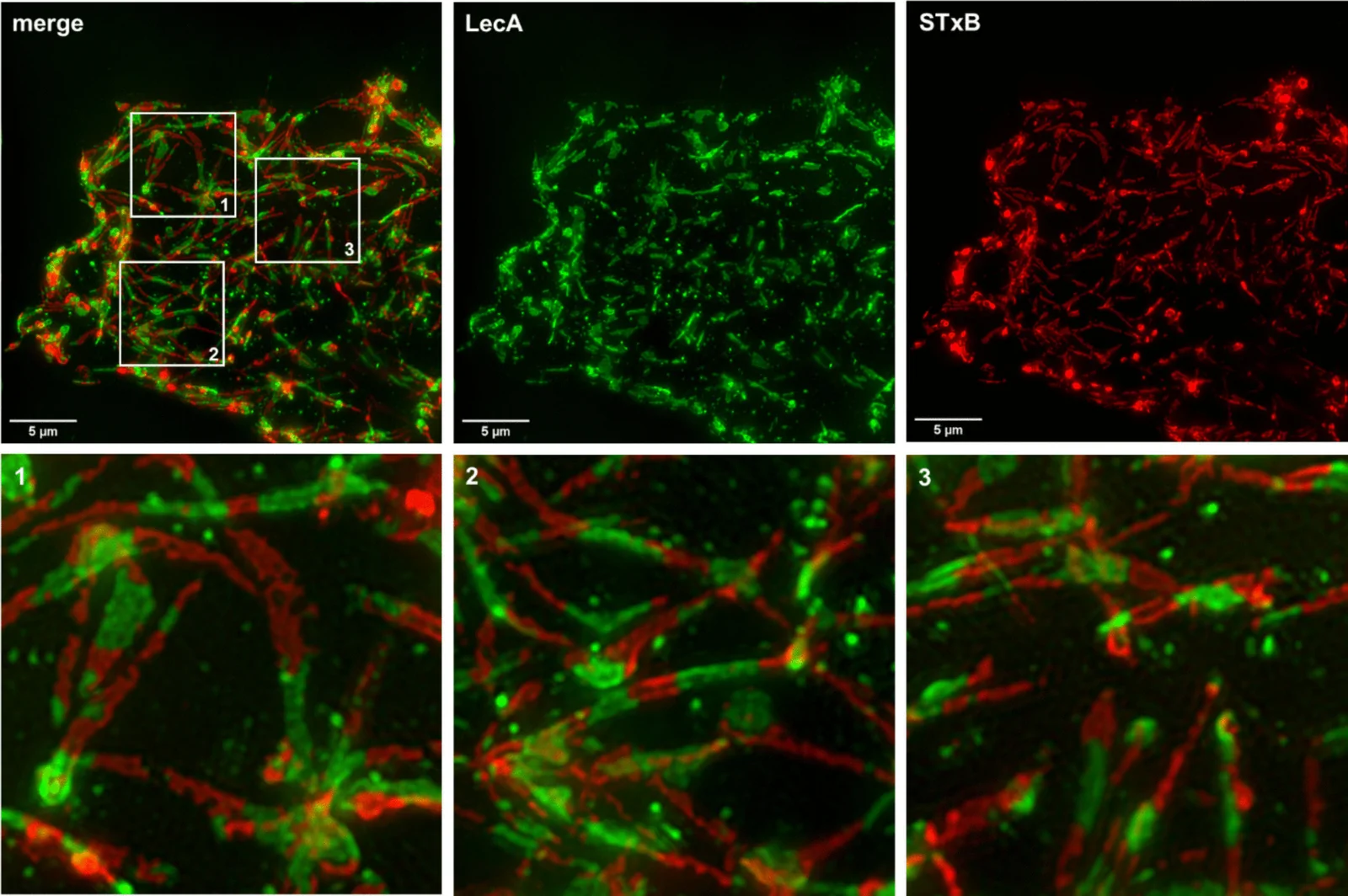
The Gb3-binding lectins LecA and STxB localize to distinct sections of the same tubular structures resolved by structured illumination microscopy (SIM). The resolution of SIM enables the detection of domains and structures in the sub-micrometer range. HeLa cells were energy-depleted and then incubated with LecA-Alexa Fluor 488 (250 nM, green color) and STxB-Cy3 (200 nM, red color) for 15 min at 37 °C. Samples were imaged using a Nikon-N-SIM microscope equipped with laser lines at 488 nm and 561 nm, and a CFI apochromatic 100× oil immersion objective (NA 1.49). Scale bars: 5 µm

The interactions of the glycosphingolipid Gb3 with the B-subunit of Shiga toxin from *Shigella dysenteriae* constitute the first step of toxin uptake into cells and form the basis of an alternative mechanism of clathrin-independent endocytosis. STxB, which can recruit up to 15 Gb3 molecules under its pentameric structure, can induce the formation of narrow tubular invaginations on the plasma membrane of target cells through the clustering of Gb3, without requiring cytosolic factors. The length and number of inward-facing tubules increased with energy depletion or inhibition of dynamin and actin, suggesting that the activity of cellular factors is only necessary for subsequent scission of tubular structures from the cell membrane, but not for the invagination process. By reconstituting the process of tubule invagination in membrane model systems, the authors showed that tubule formation was promoted by Gb3 species with long and unsaturated fatty acyl chains and prevented under high membrane tension (Römer et al. 2007, 2010). In subsequent years, this cargo-induced mechanism of clathrin-independent endocytosis via glycolipid clustering was extended to other toxins such as cholera toxin, viruses such as Norovirus (Rydell et al. 2013) and SV40 (Ewers et al. 2010), and bacteria such as *P. aeruginosa* (Eierhoff et al. 2014). Similar tubular membrane invaginations were observed as a result of interactions of the tetrameric *P. aeruginosa* lectin LecA with Gb3 in cells and model membrane systems using various microscopy techniques, including correlative light and electron microscopy (CLEM) (Darkow et al. 2020). The bacterial surface lectin LecA targets Gb3 and promotes bacterial invasion via a lipid zipper mechanism (Eierhoff et al. 2014). Furthermore, it has been demonstrated that LecA can crosslink opposing membranes of cells and GUVs due to the opposite orientation of two of its four carbohydrate binding sites (Villringer et al. 2018). On phase-separated supported lipid bilayers containing Gb3, it was found that LecA induces the dispersion of ordered membrane domains, followed by the formation of multilayers and the disintegration of the membrane. Of note, using the same type of membranes in combination with confocal microscopy, the bacterium *P. aeruginosa* mostly bound to the interphase of liquid-ordered (Lo) and liquid-disordered (Ld) membrane domains. Simultaneously with the formation of bacterial aggregates and the accumulation of membrane lipids, the lipid bilayers were drastically reorganized and Lo domains were dissolved (Stober et al. 2025). When incubated simultaneously with energy-depleted HeLa cells, structured illuminated microscopy showed that LecA and STxB were localized to the same tubular structures but mainly to different membrane nanodomains even though they shared the glycosphingolipid Gb3 as a receptor (Fig. 2).

Moreover, STxB recognized Gb3 at the primary cilium and the periciliary membrane of polarized cells, whereas LecA only bound to periciliary Gb3 (Schubert et al. 2020). By reconstituting lectin-Gb3 interactions using giant unilamellar vesicles, LecA and STxB domains could indeed be separated under specific conditions. Both the Gb3 fatty acyl chain structure and the local membrane environment modulated Gb3 recognition by LecA and STxB, although several Gb3 species were bound equally by both lectins (Schubert et al. 2020). Taken together, these studies have consistently demonstrated that multivalent lectins induce lipid relocation in the membrane and affect local membrane curvature. The selectivity of Gb3-binding lectins towards membrane domains containing different GSL species (differing in the fatty acyl chain length, hydroxylation and degree of saturation) still remains unclear. The topology of the individual carbohydrate binding site and their mutual orientation on the lectin surface seem to be important, as reviewed in Schubert and Römer (2015).

### Visualization of membrane dynamics in the presence of gangliosides

Direct observation of the accumulation of the ganglioside GM1, but not Gb3, during the uptake of the bacterium *Neisseria meningitidis* in human brain microvascular endothelial cells was revealed by SIM and direct STORM (dSTORM). In contrast, corresponding dSTORM images without bacteria showed a more homogeneous distribution of GM1 and Gb3 without clear signs of clustering, supporting the bacteria-induced GSL clustering mechanism (Schlegel et al. 2019).

Other studies revealed that GSL clustering results in a transient coupling of the two membrane leaflets. High-speed single-particle tracking using an interferometric scattering microscopy, in which molecules labeled with 20–40 nm scattering gold beads were tracked and revealed the molecular pinning of GM1 bound to CTxB. The interleaflet coupling between lipid tails at the nanoscopic scale was sufficient to induce transient immobilization and thus anomalous sub-diffusion in the millisecond range (Spillane et al. 2014). The re-localization of GM1 during membrane remodelling by sphingomyelinase, which hydrolyzes sphingomyelin to ceramide, was visualized in real-time using fluorescently labeled CTxB binding to GM1. Two types of GM1 clustering were observed: firstly, rapidly formed and short-lived clusters of GM1 in ceramide-rich domains nucleating from the liquid-disordered phase; and secondly, late-formed and long-lived high-density GM1 clusters formed in the liquid-ordered phase (Lee and Choi 2021). On the other hand, the clustering of ganglioside molecules on the surface of living cells by multivalent murine polyoma virus-like particles (VLPs) and transmembrane coupling were assumed to be the cause of the confinement of the virus-receptor complex by cortical actin filaments (Ewers et al. 2005). Similar to the interactions of STxB with Gb3 (see above), Simian virus 40 and its capsid protein VP1, which binds to GM1, induced negative membrane curvature and tubular membrane invaginations in both cellular plasma membranes and GUVs. The formation of membrane invaginations required long GM1 fatty acyl chains, whereas short chains were unable to support invagination or subsequent endocytosis and infection (Ewers et al. 2010). Furthermore, the visualization of SARS CoV-2 entry using STORM showed that it was regulated by cholesterol-assisted clustering of ACE2 in nanodomains containing GM1; ACE2 moved to GM1 clusters when the cholesterol level was high (Wang et al. 2023). These experimental findings provide evidence from different perspectives of complex, pathogen-induced membrane dynamics supported by various molecular players, implying an important role of GSL species in these processes.

Further direct illustration of the importance of the ceramide structure for GM1 clustering was demonstrated using STORM and fluorescence emission anisotropy measurements, which detects fluorescence resonance energy transfer (FRET). The exogenous fluorescently labeled GM1 molecules incorporated into the plasma membrane of living cells formed nanodomains when they had a C16:0 fatty acyl chain, but not when they had a C16:1 fatty acyl chain (Arumugam et al. 2021). Furthermore, taking into account the presence of phosphatidylserine (PS) and the decoupling of the acto-myosin machinery in cell-derived giant plasma membrane vesicles (GPMVs or blebs), the authors demonstrated that the active clustering of the Alexa Fluor 488-GM1 C16:0 species depends on membrane cholesterol, actin, and PS. The binding of CTxB led to enlarged membrane domains for both C16:0 and C16:1 fatty acyl chains, but only the C16:0 fatty acyl chain favored the co-distribution with the GPI-anchored protein CD59. In the absence of CTxB, the CD59-GM1 clusters were not detected by STORM on fixed cells (Arumugam et al. 2021).

In the absence of bacteria or their lectins, a study using single-molecule imaging and tracking with total internal reflection fluorescence (TIRF) microscopy in plasma membranes of living cells also showed clear but transient co-localization and co-diffusion of fluorescent ganglioside analogues of GM1 and GM3 with a fluorescently labeled CD59. Co-distribution was observed with lifetimes of 12 ms for CD59 monomers, 40 ms for transient CD59 homodimer rafts in quiescent cells, and 48 ms for engaged-CD59-cluster rafts, depending on cholesterol and GPI-anchoring. The ganglioside molecules were always mobile and exchanged continuously and dynamically between the raft domains and the bulk (Komura et al. 2016). Furthermore, single-molecule imaging with enhanced temporal resolution of up to 5 ms revealed that the stably induced clusters between CD59 and GM1 in the outer leaflet of the plasma membrane continuously recruited Lyn and H-Ras directly beneath them in the inner leaflet with dwell lifetimes of < 100 ms (Koyama-Honda et al. 2020). Following live diffusion of fluorescent GM1 and an Atto 647N-labeled GPI-anchor using STED microscopy in combination with fluorescence correlation spectroscopy (FCS), the transient trapping of sphingomyelin, GM1 and the GPI-anchor in cholesterol-mediated molecular complexes within < 20 nm in the membrane of living mammalian cells was demonstrated (Eggeling et al. 2009). Furthermore, STED-FCS confirmed the trapped-like diffusion and transient nanodomain incorporation of the ganglioside GM1 in both the membranes of living cells and GPMVs (Schneider et al. 2017). The interactions of GM1 have been described not only for raft-associated GPI-anchored proteins, but also for other signalling proteins. For example, GM1-enriched domains can form in response to a transmembrane protein, EphA2 receptor tyrosine kinase, through oligomerization by its ligand ephrinA1-Fc. The GM1-enriched microdomains formed concomitantly with EphA2-enriched microdomains (Wirth et al. 2022). These experiments provide insight into the heterogeneity of the plasma membrane, which goes beyond the spatiotemporal properties of molecular interactions that can be captured using fluorescent imaging techniques.

Taken together, the results obtained using microscopy techniques confirm both the spontaneous transient phenomena of co-occurrence of GSLs and signalling proteins in nanoscale domains of the plasma membrane and the lectin-induced GSL clustering, lipid reorganization and membrane bending depending, amongst others, on the structure of the fatty acyl chain.

## Computer simulations

Molecular dynamics (MD) simulation is a highly complementary approach to microscopy. Over the decades, it has matured into a tool that functions conceptually like a computational microscope (Dror et al. 2012; Stevens et al. 2023). It allows simulating molecular motions, including translational, rotational, and conformational modes of individual molecules that are affected by the molecular environment in a complex molecular system. With the advances in computer power, including the application of graphical processing units (GPUs), the complexity and the spatiotemporal scales of MD simulations have increased remarkably, now reaching systems with hundreds of millions of atoms (Stevens et al. 2023).

Multiscale computer modelling offers a range of intrinsically consistent approaches for extending the spatio-temporal framework of simulated events with adequate computer power (Perez et al. 2021) (Fig. 1). It is based on a step-by-step simplification of the description of the atomistic and sub-atomistic architecture and thus on an acceleration of the calculations of a reduced number of degrees of freedom in the molecular system. At the same time, there is always a possibility to map the system back to a more detailed molecular representation (López et al. 2022). Coarse graining (CG) is a way of simplifying the all atom (AA) presentation by grouping several heavy atoms in one pseudo-atom. The loss of directionality of hydrogen bonds limits the calculation of glycolipids to a certain extent, but modern force fields attempt to solve this problem (Souza et al. 2021). The temporal scales in computer simulations range from the highest vibrational frequencies, corresponding to the stretching of the shortest covalent bonds in molecules (with characteristic times of ~10 fs), to the total duration of the trajectory, which depends on the available computing power but is usual of the order of µs. With respect to glycolipids, important features that MD can access are the conformational dynamics of glycolipids and short-lived lipid clusters. The conformational flexibility of carbohydrates is the result of various relaxation processes, including interconversion between various ring puckering transitions (~100 ns), the rotation of the primary and secondary hydroxyl groups (from ps to ns), and the rotation time around glycosidic linkages (~10 ns) (Kuttel 2011). Lipid clustering events can be observed over tens to hundreds of nanoseconds of the CG MD trajectory (Nitenberg et al. 2020; Makshakova et al. 2020) due to the smoothening of the energy landscape compared to AA simulations (Perez et al. 2021).

Gangliosides, in particular GM1, were the major glycolipids to date used to investigate the driving forces of lipid clustering in membranes *in silico*. AA simulations showed that in a lipid bilayer consisting mainly of DOPC molecules, the GM1 headgroup interacts with the choline group of the DOPC molecules with a lifetime of a few ns, which is much longer than the interactions between DOPC molecules themselves (Sega et al. 2006). Furthermore, the GM1 molecules in the DOPC membrane existed in two equilibrium states that differed in the orientation of the branched headgroup (Jedlovszky et al. 2009). Nonetheless, it was found that the conformation of the headgroup of GM1 was very similar in liquid-ordered and liquid-disordered phases (Rissanen et al. 2017). In addition, simulations revealed nanoscopic segregation of gangliosides, which was favored by the self-assembly of their headgroups with a domain radius between 7 and 120 nm (Sarmento et al. 2021). Energy estimations showed that the ganglioside headgroups contribute up to 67% of the total interaction energy between gangliosides due to the formation of a hydrogen bond network. However, the N-acetyl galactosamine moiety of ganglioside GM2 disrupts this network (Sarmento et al. 2021). On the other hand, the smaller headgroups of GM3 allowed for slightly larger and more ordered clusters than those of GM1 (Gu et al. 2017). Sialic acid residues stabilize the hydrogen bond network between gangliosides (with ∼6.2 bonds), and promote the formation of nanodomains even without cholesterol or sphingomyelin. Without sialic acid residues, such as in asialo-GM1, the number of hydrogen bonds decreased significantly (~2.8 bonds) (Davidović et al. 2023). Coarse-grained (CG) simulations of lipid bilayers mimicking neuronal plasma membranes reproduced the importance of hydrogen bonding between GM1 (with 18:0 acyl chain) molecules resulting in the formation of larger clusters that led to increased ordering and reduced membrane fluidity (Lyu et al. 2024). A series of AA and CG simulations confirmed the interdigitation of the long fatty acyl chains of GM1 in the extracellular membrane leaflet with the opposite membrane leaflet and the change of the phase behavior in the cytosolic, GM1-free, leaflet. Short-chain GM1 species maintained phase-symmetric bilayers with a membrane registration effect, whereas long-chain GM1 molecules were significantly interdigitated with the lipids of the cytosolic leaflet resulting in weakening and slowing down of the membrane registration process (Manna et al. 2017). Another, even less explored aspect is how the fatty acyl chains and headgroups of GSLs influence the localization, clustering, binding and conformation of proteins. For the glycagon receptor, which belongs to G-protein-coupled receptors, multiscale MD simulations disclosed that movements of its extracellular domain between open and closed conformations in respect to the transmembrane domain were modulated by the binding of GM3 in the presence of native-like membrane bilayers (Ansell et al. 2020). Ganglioside enrichment around membrane-bound proteins under conditions mimicking the plasma membrane, a bilayer of 60 types of lipids, was investigated using CG MD and ten different signalling proteins (Corradi et al. 2018). However, the results depended on the water model used, which made the interpretation of the results of modelling difficult without experimental benchmarks.

**Fig. 3:**
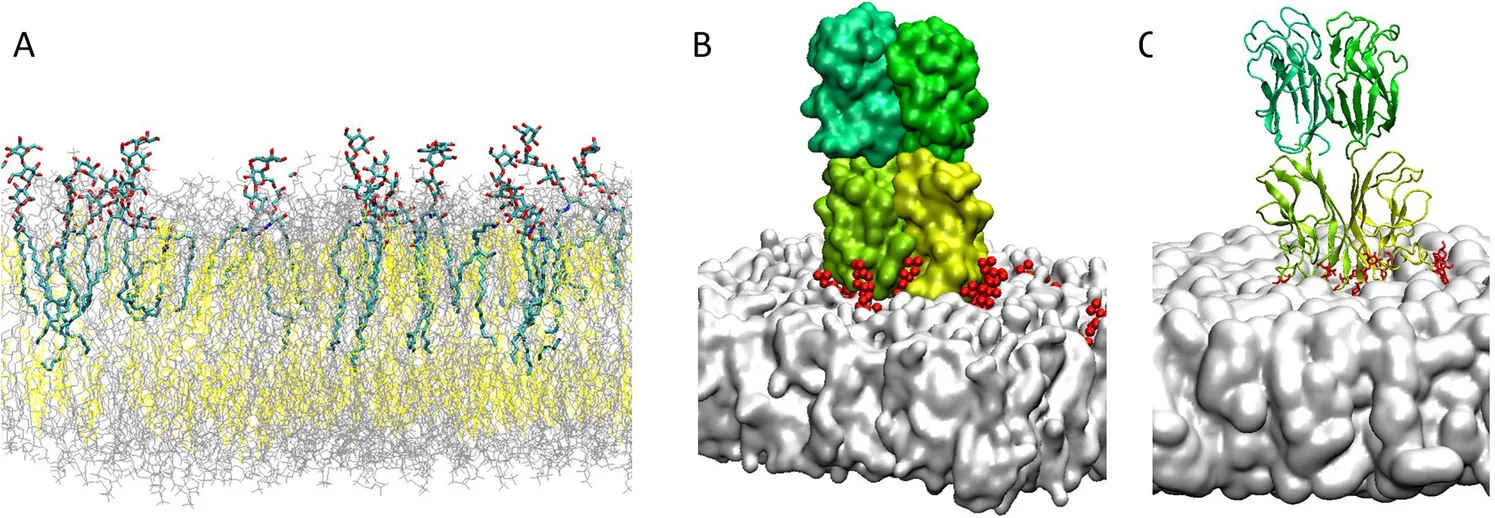
Interactions of the *P. aeruginosa* lectin LecA with the glycosphingolipid Gb3 in a lipid bilayer. **A** All-atom presentation of a Gb3-containing lipid bilayer consisting of DOPC/cholesterol/Gb3 (65/30/5 mol-%). The lipids are shown in grey (DOPC), yellow (cholesterol) and cyan (Gb3), respectively. The oxygen atoms of Gb3 are highlighted in red, its nitrogen atoms in blue. **B** Side view of LecA bound to the Gb3-containing membrane and equilibrated in the course of coarse-grained MD. The membrane is shown as gray surface, the four LecA monomers are shown as surface in yellow to green colors, the pseudo-atoms of Gb3 are presented as red spheres. **C** The back-mapped and refined all-atom representation of LecA bound to the Gb3-containing membrane. The color-coding is similar to panel B. The membrane is shown as grey surface, the LecA monomers as yellow to green cartoons, Gb3 is given in sticks representation

The clustering of glycolipids beneath multivalent lectins and subsequent changes of membrane properties was a subject of several studies. AA MD simulations revealed that GM1 with unsaturated fatty acyl chains formed clusters through the binding of CTxB, which were less ordered compared to clusters with saturated GM1 species (Arumugam et al. 2021). The GSL clustering resulted in membrane bending. With increasing GM1 concentrations in a POPC bilayer, tight lipid packing driven mainly by carbohydrate-carbohydrate interactions led to a preference for positive membrane curvature and for larger cluster sizes, as revealed by AA simulations (Patel et al. 2016). Furthermore, the membrane bending as an initial phase of the cellular uptake of viral and bacterial lectins was addressed by AA, CG, and mesoscale simulations (see reviews (Pezeshkian and Marrink 2021; Pezeshkian et al. 2021) and references therein). AA MD studies investigated the ability of CTxB (Pezeshkian et al. 2017) and STxB (Pezeshkian et al. 2016) to induce negative membrane curvature due to their multivalent binding to up to five and fifteen GSLs, respectively. In a similar way, the pentameric VP1 capsid protein of SV40 clustered GM1 molecules as revealed by CG MD (Kociurzynski et al. 2019). The binding of the *P. aeruginosa* lectin LecA to Gb3 on DOPC/cholesterol lipid bilayers also caused the GSL molecules to group together in a larger cluster beneath the protein surface, even though only two Gb3 binding sites of LecA were exposed to the membrane surface (Fig. 3). The clustering promoted local negative curvature of the lipid bilayer (Kociurzynski et al. 2021).

## Cross‑validation between simulations and experiments

Although there is still a gap between the spatial and temporal resolution achieved by fluorescence microscopy and computer modelling, both come fairly close under certain conditions.

In general, the microscopy experiments deal with larger scales and observe the co-occurrence of molecules, which is visible as an overlap of fluorophore signals. Given the currently available resolution of imaging techniques, no clear conclusions can be drawn about direct interactions between these molecules. Computer modelling, in turn, can take the co-occurrence of molecules as a starting assumption for possible interactions and confirm or refute direct interplay at the molecular level. Furthermore, modelling based on an atomistic model of interactions in a membrane can suggest mutations or inhibitors to disrupt or stabilize molecular interactions, which can be confirmed experimentally.

**Fig. 4:**
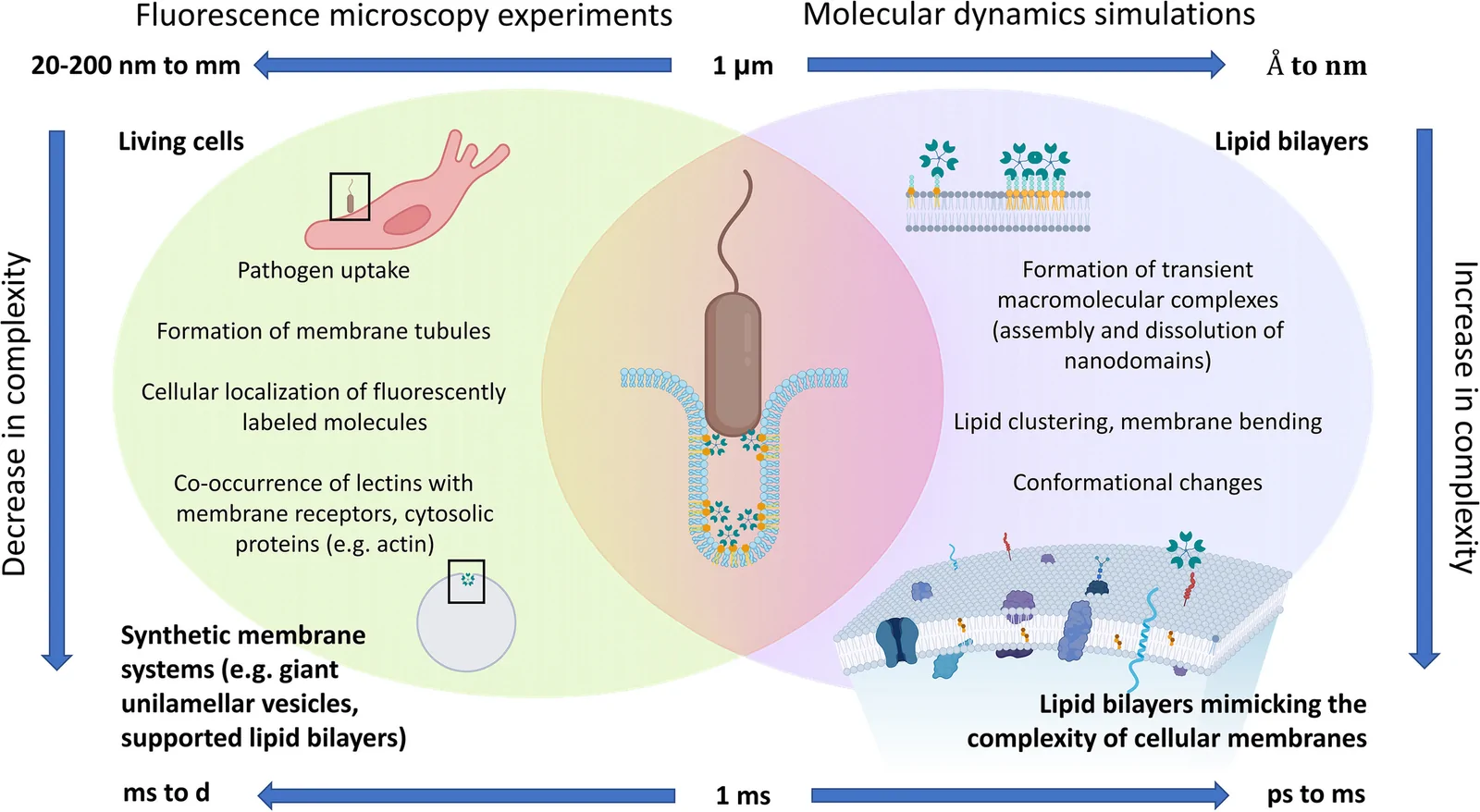
Cross-validation of experiment and simulation. Schematic representation of diverse GSL-containing membranes of different complexity (e.g., cell membranes, synthetic membrane systems, and *in silico* lipid bilayers) and lectin-induced membrane processes (e.g., clustering, membrane bending and invagination) observed using fluorescence microscopy and/or computer modelling. The lectin-induced GSL clustering, subsequent membrane bending and formation of tubular membrane invaginations are at the interface of the spatial (upper horizontal arrows) and temporal (lower horizontal arrows) resolutions of the two approaches. The figure elements were created by BioRender

The example shown in Fig. 4 can serve as cross-validation between simulation and experiment. To better understand the mechanism of lectin-induced formation of tubular membrane invaginations, which could be observed on cells and synthetic membrane systems using microscopy techniques, computer modelling was crucial for elucidating lectin-triggered GSL clustering, the dynamics of other membrane lipids and the initial inward bending of the membrane.

Another example illustrating how modelling and experiments inform each other in a continuous line of research was briefly discussed in the experiments section. Microscopy studies have shown that the C16:0, but not the C16:1 fatty acyl chain of GM1 favored its co-occurrence with CD59 upon CTxB binding. Subsequent AA MD simulations explained this finding by showing that the acyl chain of unsaturated GM1 becomes more disordered upon CTxB binding compared to the saturated GM1 chains, such that increased C16:1 disorder can exclude CD59 from association with the lectin-induced domains (Arumugam et al. 2021).

Although modelling a larger and more complex membrane fragment containing various lipids including GSLs and proteins would be extremely valuable, the relaxation time of the modelled system must be taken into account as a limitation.

## Future perspectives

In order to outline the future prospects for GSL biology and the necessary methodological advances, the most important open questions can be summarized as follows: (1) How do diverse carbohydrate headgroups and lipid tails of GSLs influence their presentation on the plasma membrane and thus their recognition? (2) What are the molecular mechanisms that drive the incorporation or exclusion of GSLs into membrane nanodomains, and how do they modulate cellular processes? (3) What is the composition of GSL-containing signalling complexes? (4) How do GSLs influence host-microbe interactions, malignancy progression, and immune responses?

To further understand these mechanisms, biochemical and biophysical approaches must go hand in hand. On the biophysical side, integrated imaging and modelling platforms are needed to link the spatial organization of GSLs at the molecular level to real-time cell signalling and trafficking. In addition, predictive computational frameworks for interpreting how changes in GSL conformation affect membrane biophysics and cellular networks will be of great help.

Despite major successes in revealing the distribution of membrane components and their pathogen-induced redistribution during signal transduction using fluorescence microscopy techniques, the experimental ability to detect complex formation lies beyond spatial and/or temporal resolution. The emergence of new high-resolution microscopy techniques promises more detailed insights into the structure and dynamics of GSL-associated signalling complexes in the plasma membrane. Among the noteworthy methods, MINimal emission FLUXes (MINFLUX) stands out, combining rough confocal Gaussian beam mapping with switching to excitation by a donut beam (Salerno et al. 2025) and achieving exceptional localization accuracy of up to 2 nm (Balzarotti et al. 2017; Moosmayer et al. 2024). However, to increase tracking speed, the accuracy of localization tracking was reduced to ~ 20 nm (Salerno et al. 2025; Moosmayer et al. 2024; Schmidt et al. 2021). Points Accumulation for Imaging in Nanoscale Topography (PAINT) is a variation of SMLM, which uses fluorescent probes that only emit signals when transiently bound to specific target molecules (resolution 10–30 nm). This method was applied to study single carbohydrates of glycans in their natural environment. DNA origami (Reinhardt et al. 2023) or lectins (Riera et al. 2021; Tholen et al. 2025) can be used as probes. The transient nature of the binding leads to an apparent blinking of the target, necessary to perform SMLM. When nanobodies conjugated with DNA-barcodes were used for a target molecule recognition, the SMLM Resolution Enhancement by Sequential Imaging (RESI) achieved 5 nm resolution (Reinhardt et al. 2023). The size limitation from using nanobodies was overcome with the combination of RESI and bioorthogonal metabolic labeling enabling Ångström-resolution (< 1 nm) mapping of glycans (sialic acid was used as a proof-of-concept) in fixed cells with TIRF microscopy (Masullo et al. 2025). Lectin-PAINT, which uses weak reversible lectin binding to glycoconjugates to visualize the cell glycan fingerprint, was enabled at a single-molecule level in living cells (Riera et al. 2021; Tholen et al. 2025).

Given that computer resources are constantly increasing, there are fewer and fewer limitations to target the dimensions of the entire cell with modelling (Stevens et al. 2023). However, capturing the temporal aspects of the diffusion of membrane components with the formation of transient signalling complexes remains challenging. Multiscale modelling provides a conceptual platform for accelerating calculations by omitting some molecular details and reconstructing them when necessary. To make the transitions between different levels of multiscale modelling efficient, an on-the-fly back mapping procedure like Multiscale Machine-Learned Modelling Infrastructure (MuMMI) (Ingólfsson et al. 2023) is required. On the other hand, there is still a need to develop more accurate force fields, whereby machine-learning and deep-learning approaches can be helpful (Unke et al. 2021).

Despite all this, the main difficulty for meaningful modelling still remains the lack of understanding of how the plasma membrane is organized at a molecular level at sites of emerging signals. Modelling requires a deeper understanding of the interacting molecular partners derived from experiments, including the precise characteristics of the fatty acyl chain of the GSL. Together, fluorescence microscopy and computer modelling can provide a holistic view of the functionality of GSLs in membrane nanodomains, thereby bridging the gap between details at the molecular scale and processes at the cellular level.

These methodological advances will not only be useful to improve our fundamental understanding of cell signalling mechanisms and how pathogens decipher the host “glycocode”, but will also have a major impact on the development of therapeutic applications such as cancer diagnostics, pathogen targeting and drug delivery. Understanding the formation of transient nanodomains and their composition at the molecular level, the presentation of GSLs on the membrane surface in this context, the main triggers of membrane interleaflet coupling and transmembrane signalling will help to develop next-generation molecular diagnostics and targeted delivery tools such as CAR-T cells or lectin-antibody chimeras. This understanding will support the knowledge-based design of applications of practical relevance based on natural or modified lectins, peptides, RNA-based aptamers or synthetic binders, which together will provide tools for modulating cell-cell communication with great potential value.

## Data Availability

No datasets were generated or analysed during the current study.
